# Facilitating model reconstruction for single-particle scattering using small-angle X-ray scattering methods^1^

**DOI:** 10.1107/S1600576716004337

**Published:** 2016-03-18

**Authors:** Shufen Ma, Haiguang Liu

**Affiliations:** a Complex Systems Division, Beijing Computational Science Research Centre, 8 W Dongbeiwang Road, Beijing, 100193, People’s Republic of China

**Keywords:** X-ray free-electron lasers, XFELs, small-angle X-ray scattering, protein structure, single-particle imaging, computer programs

## Abstract

X-ray free-electron lasers generate intense femtosecond X-ray pulses, so that high-resolution structure determination becomes feasible from noncrystalline samples, such as single particles or single molecules. At the moment, the orientation of sample particles cannot be precisely controlled, and consequently the unknown orientation needs to be recovered using computational algorithms. This delays the model reconstruction until all the scattering patterns have been re-oriented, which often entails a long elapse of time and until the completion of the experiment. The scattering patterns from single particles or multiple particles can be summed to form a virtual powder diffraction pattern, and the low-resolution region, corresponding to the small-angle X-ray scattering (SAXS) regime, can be analysed using existing SAXS methods. This work presents a pipeline that converts single-particle data sets into SAXS data, from which real-time model reconstruction is achieved using the model retrieval approach implemented in the software package *SASTBX* [Liu, Hexemer & Zwart (2012). *J. Appl. Cryst.***45**, 587–593]. To illustrate the applications, two case studies are presented with real experimental data sets collected at the Linac Coherent Light Source.

## Abstract 2

This article describes software for fast model reconstruction from small-angle scattering data, providing real-time feedback on experiments.

## 1. Introduction   

One of the ultimate goals for the structural biology community is to study molecular structure and dynamics using single molecules as samples. Heretofore X-rays have been widely applied in structure determinations of matter at microscopic scales. Because of the radiation damage induced by X-rays, the dosage has to be limited to ensure that the sample is not destroyed during measurement. To obtain high-resolution signals, photons scattered from identical copies that are arranged in periodic lattices are summed: this is the approach adopted in X-ray crystallography. For noncrystalline samples, the signals are spherically averaged, yielding a one-dimensional scattering profile that contains structural information. This is the so-called small/wide-angle X-ray scattering (SAXS/WAXS) method, which is a powerful technique for studying molecular structure and dynamics in solution (Datta *et al.*, 2009 ▸; Hura *et al.*, 2009 ▸; Putnam *et al.*, 2007 ▸; Nishimura *et al.*, 2009 ▸).

It has been predicted that single-molecule structure determination will be feasible using ultrashort X-ray pulses, which can outrun radiation damage (Neutze *et al.*, 2000 ▸). The commissioning of the hard X-ray free-electron laser (XFEL) at the Linac Coherent Light Source (LCLS) in the Stanford Linear Accelerator Center excited the structural biology community, as the ultimate goal is one step closer (Emma *et al.*, 2010 ▸). At LCLS, XFEL pulses are generated at 120 Hz, with each pulse enclosing over 10^12^ photons, and the pulse duration can be as short as a few femtoseconds. More importantly, the X-rays are transversely coherent and can be focused onto a region with a radius as small as 100 nm. This super brightness enables sufficient photons to be scattered from a single particle (such as a molecule) to attain high-resolution signals in each scattering pattern. After measuring many scattering patterns, each from a fresh sample particle at an unknown orientation, the scattering intensity distribution in reciprocal space can be obtained if the orientation distribution covers SO3 space. However, because the interaction volume of the X-rays and particles is very low for the current experimental setup, the chance of a particle meeting X-ray pulses at the focus is extremely small (progress has been made to improve the chance of particles being intercepted by X-ray pulses). In most cases, the particles are actually in the region away from the X-ray focus where the incident X-ray intensity is relatively low, resulting in low scattering intensity, especially at large scattering angles. Furthermore, because the samples are injected into the experimental chamber, there is no reliable control of the particle orientations. To merge the intensity of individual scattering patterns in three-dimensional reciprocal space, the particles and their associated patterns have to be re-oriented first. In principle this can be achieved by studying the relationships between scattering patterns or comparing the patterns against templates generated from a reference model. There are several algorithms that have been proposed to address this issue (Bortel & Tegze, 2011 ▸; Fung *et al.*, 2009 ▸; Loh & Elser, 2009 ▸; Kassemeyer *et al.*, 2013 ▸). For example, the expansion–maximization–compression algorithm and the geodesic distance based algorithm have demonstrated applicability in real experimental data analysis (Loh & Elser, 2009 ▸; Ekeberg *et al.*, 2015 ▸; Kassemeyer *et al.*, 2013 ▸). The recovery of orientation information is critical and often takes several rounds to achieve convergence. To provide real-time feedback with reconstructed models, other approaches have to be developed besides the single-particle imaging strategy (*i.e.* re-orienting and merging of the single-particle scattering data in the three-dimensional scattering volume for iterative phasing).

One alternative approach is to represent the structural information in relative coordinates, similar to the form of Patterson functions. This technique is called fluctuation scattering or fast solution scattering, in which the correlations between intensities at different reciprocal coordinates are calculated from raw scattering patterns (Kam, 1977 ▸; Saldin *et al.*, 2010 ▸). If the orientations of the measured particles are random, the intensity correlation functions converge to the correlation function of a single particle, as shown by Kam (1977 ▸). In other words, the correlation function is another representation of intensity distribution, with parameters of relative coordinates instead of absolute Cartesian coordinates, and such coordinates are independent of orientation. Algorithms have been developed for model reconstructions based on these correlation functions or their derivatives (Saldin *et al.*, 2011 ▸; Liu *et al.*, 2013 ▸; Donatelli *et al.*, 2015 ▸).

Here, we present an approach for quick model retrieval from a SAXS profile obtained by merging single-particle scattering data. As stated previously, despite the advances of model reconstruction algorithms, both the single-particle imaging approach and the correlation scattering approach take substantial time and effort before a reasonable three-dimensional model can be built. Given the fact that an ensemble of single-particle scattering patterns can be summed to form a ‘virtual powder diffraction pattern’ or ‘virtual SAXS pattern’, we can borrow the methods of SAXS, which is a mature experimental method to study noncrystalline samples in bulk (Glatter & Kratky, 1982 ▸). The data analysis and model reconstruction tools are well developed: for example, the *ATSAS* package developed at EMBL (Petoukhov *et al.*, 2007 ▸) and the *SASTBX* package (Liu, Hexemer & Zwart, 2012 ▸). In spite of the low information content of the one-dimensional SAXS curve, some important information can be extracted, such as molecular weight, radius of gyration, fractal properties and so on. In this work, we will demonstrate how SAXS methods can facilitate single-particle scattering data analysis during XFEL experiments and how real-time feedback with three-dimensional models can be possible using the fast model retrieval methods implemented in *SASTBX* (Liu, Hexemer & Zwart, 2012 ▸). A demonstration of fractal morphology analysis using SAXS methods for single-particle scattering experimental data can be found in the work by Loh *et al.* (2012 ▸).

The challenge of model reconstruction from a SAXS profile is mainly due to the limited information contained in the one-dimensional profile. The intrinsic deficiency of this model reconstruction can be partially compensated for by constraints from other sources, such as knowledge about the model in real space (whether the object is isolated in space, positivity, continuity *etc.*), which is the approach taken by programs in *ATSAS* (Svergun, 1999 ▸; Svergun *et al.*, 2001 ▸; Franke & Svergun, 2009 ▸). Otherwise, only low-resolution models corresponding to a molecular envelope can be obtained using the analytical approach with parameter optimization methods (Svergun & Stuhrmann, 1991 ▸; Shneerson & Saldin, 2009 ▸). A database search algorithm has been implemented in *SASTBX* as *sastbx.shapeup*. Each SAXS profile is treated as ‘keywords’ or ‘features’, and the best matched models can be retrieved from databases that are precompiled from existing molecular structure databases. This idea of model matching is not new, and there have been some previous efforts in searching structural neighbours using SAXS profiles (Sokolova *et al.*, 2003 ▸). What distinguishes the new method, *sastbx.shapeup*, from the existing programs is that the models in the database can be scaled to any size, in order to optimize the goodness of fit to the SAXS profile, making the model radius, *r*, an extra independent parameter in the model search process. The basic principle of the *sastbx.shapeup* method was outlined by Liu, Hexemer & Zwart (2012 ▸) and a detailed explanation of the algorithm will be provided in another work. The performance of the program and the applications in XFEL-based single-particle scattering experiments are discussed below.

## 2. Methods   

The scattering data subjected to this method are limited to the small-angle regime at the moment. In LCLS experiments, the back detector (in the case where two detectors are deployed) measures the desired data, which are the focus for the analysis presented here.

### 2.1. Data analysis pipeline   

**Figure 1:**
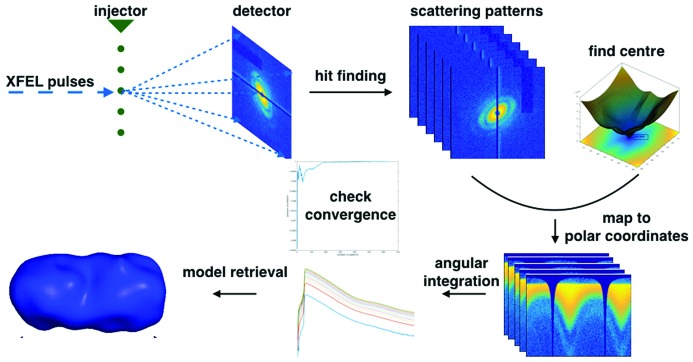
Pipeline for XFEL single-particle scattering data analysis using the SAXS approach.

The raw scattering patterns are collected at 120 Hz at LCLS at a normal repetition rate, and the scattering patterns resulting from sample particles have to be selected from a large data set using hit-finding programs such as *Cheetah* (Barty *et al.*, 2014 ▸), *OnDA* (Mariani, 2016 ▸; Mariani *et al.*, 2016 ▸) and *Hummingbird* (Daurer *et al.*, 2016*a* ▸,*b* ▸). Then the scattering centre can be optimized using Friedel symmetry at a flat region of the Ewald sphere. It is known that, near the origin of reciprocal space, the Ewald sphere can be treated as approximately flat. Without loss of generality, we define the ‘flat region’ as the region where there is less than 1% deviation of the Ewald sphere from the tangent plane perpendicular to the incident X-ray beam, and the corresponding scattering angle is about 0.07 rad. The data falling within this scattering angle can be used for scattering centre optimization, which is carried out by a grid search around the initial centre by minimizing the difference between the intensities of Friedel pairs. The centred scattering pattern is then converted to polar coordinates to obtain an ‘intensity profile’ along the radial direction by integrating the intensity over the azimuthal angle. The intensity profiles will be summed to form a SAXS profile. If the orientations of the particles that result in scattering patterns are randomly distributed, the summed intensity profile will be effectively a SAXS profile. The convergence can be monitored by comparing the Pearson correlations of the cumulative ‘intensity profile’ with any reference profile (such as the average ‘intensity profile’ of the whole data set) or monitoring the changes of accumulated profiles. This pipeline for data analysis is summarized in Fig. 1 ▸.

### 2.2. Model retrieval   

Once the ‘intensity profile’ has converged to the SAXS profile, the *sastbx.shapeup* program can be used to retrieve models that match the experimental data.

A model database comprising 10 733 models was compiled, based on the Protein Interface, Surface and Assembly (PISA) database (Krissinel & Henrick, 2007 ▸). An auxiliary program, *build_db.py*, is provided for users to compile a model database from structure collections such as the Protein Data Bank, CATH, SCOP or any other three-dimensional shape data sets. The results presented here are all from the default database (*i.e.* PISA) included in the *SASTBX* package.

The agreement between the model profile 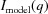 and experimental profile 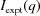 is measured by the chi score, defined as
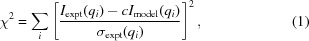
where *c* is a scaling factor obtained by least-squares minimization.

With a SAXS profile serving as the query, the *shapeup* program optimizes the radius in a range determined by the user-supplied radius 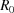 (by default, the radius searching range is from 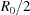 to 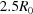), and the models with the lowest chi score are selected. For each trial radius 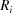, the models in the database are scaled to the same radius, and the chi score is computed for each scaled model. Using the golden section search algorithm, the radius that minimizes the chi score can be found within less than ten iterations for typical sized proteins. Then the ten models that best match the experimental SAXS profile are generated and saved in CCP4 map files.

A detailed description of the model representation using Zernike moments and computation of the SAXS profile can be found elsewhere (Liu, Hexemer & Zwart, 2012 ▸; Liu, Morris *et al.*, 2012 ▸).

## 3. Results and discussion   

### 3.1. Performance of *sastbx.shapeup* on experimental SAXS data   

**Figure 2:**
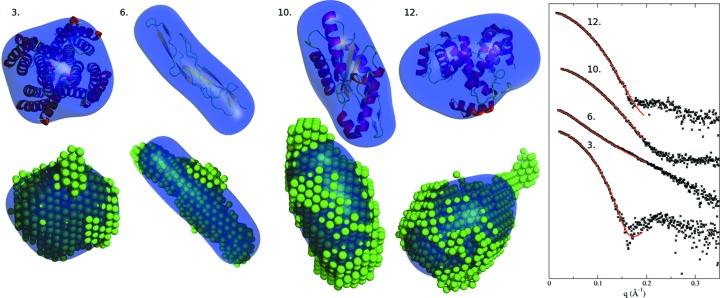
A comparison of crystal structures (top, cartoon) and *DAMMIN* bead models (bottom) with *shapeup* models (blue surface) from the data supplied by Grant *et al.* (2011 ▸) reveals a high degree of similarity between the models. The correspondence between SAXS curves from the *shapeup* models and the experimental data is shown within the *q* range that is used for *sastbx.shapeup*.

Experimental data from the BIOISIS database (http://bioisis.net; Rambo & Tainer, 2011 ▸) and data from Grant *et al.* (2011 ▸) are used as the testing data set; the accompanying models provided by experimental groups serve as reference models for structure comparison. The model retrieval execution times with default settings averaged approximately 1 min on a single 2.66 GHz CPU running Fedora 10, with the program operating in a fully automated manner. The retrieved models were compared with the structures resolved using X-ray crystallography or *DAMMIN* models. In 49 out of 52 test cases, the models are in excellent agreement. A selection of representative examples is depicted in Fig. 2 ▸, wherein the recovered models are superposed on crystal structures or *DAMMIN* models (Svergun, 1999 ▸).

### 3.2. Application for XFEL single-particle scattering   

#### 3.2.1. Nanorice single-particle scattering   

**Figure 3:**
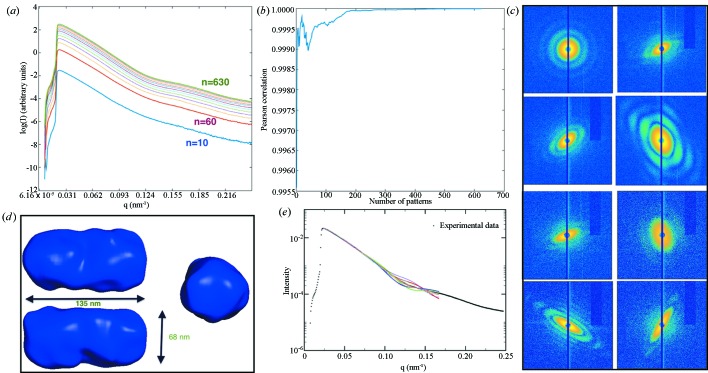
SAXS analysis for the single-particle scattering data of nanorice particles. (*a*) The progression of the virtual SAXS profile as more scattering patterns are included. (*b*) The Pearson correlation between the cumulative SAXS profile and the final SAXS profile. (*c*) Some representative single-particle scattering patterns. (*d*) The average model rendered from the top ten matched models. (*e*) The fitting of the model SAXS profiles (coloured curves) compared to the experimental data (the thicker curve).

The single-particle scattering data for iron oxide nanoparticles (Kassemeyer *et al.*, 2012 ▸) were downloaded from the Coherent X-ray Imaging database (Maia, 2012 ▸). The SAXS profile was calculated from the virtual SAXS pattern by summing 620 single-particle scattering patterns, some of which are shown in Fig. 3 ▸(*c*). Then the models that match this SAXS profile were obtained using the *sastbx.shapeup* program. The calculated SAXS profiles of retrieved models were compared with the experimental SAXS data (Fig. 3 ▸ *e*). The ten models with lowest chi scores are very consistent, and the average model is shown in Fig. 3 ▸(*d*). This model has estimated diameters of 135 nm along the long axis and 68 nm along the short axis (Fig. 3 ▸ *d*). This is in reasonable agreement with the reconstructed model reported by Kassemeyer *et al.* (2013 ▸), who recovered the orientations of the single-particle scattering patterns and merged them in three-dimensional reciprocal space to carry out a phase retrieval model reconstruction. It is encouraging that by using the spherically averaged intensity, *i.e.* the SAXS data, the *sastbx.shapeup* program can obtain similar results. This information is valuable during experiments, as we can see from Figs. 3 ▸(*a*) and 3 ▸(*b*), which show the convergence progress by monitoring the cumulative SAXS profile. For this highly symmetric object, the SAXS profile exhibited a clear converging trend after including 300 scattering patterns. The Pearson correlation is calculated with respect to the final SAXS profile obtained from all scattering patterns. The large fluctuation in the correlation curve is mainly due to false-positive single-particle scattering patterns (false-positive patterns are the patterns that result from non-sample scattering, but are mistakenly treated as sample particle scattering) that were not filtered out during the pattern selection procedure.

#### 3.2.2. Polystyrene dumbbell scattering data   

**Figure 4:**
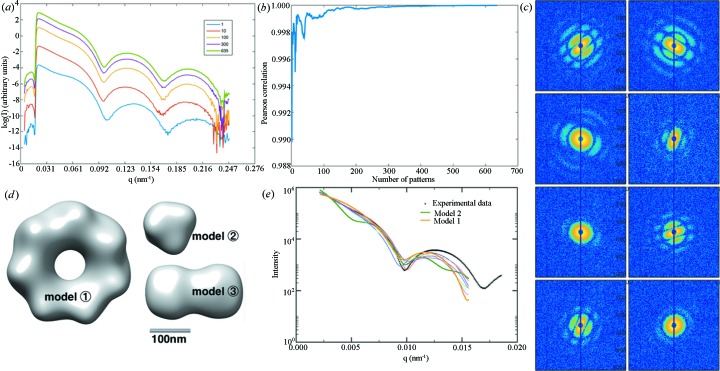
SAXS analysis for the single-particle scattering data of polystyrene dumbbell particles. The descriptions are the same as in Fig. 3 ▸, except that the *sastbx.shapeup* program retrieved three different types of model (*d*). Model 3 is consistent with the expected dumbbell shape, while the other two models might reflect aggregated (1) or dissociated (2) spheres. The SAXS curves corresponding to models 1 and 2 are indicated in (*e*), and the other eight SAXS curves all correspond to dumbbell shaped models; the average density map is represented as model 3.

In this publicly available data set, the samples are polystyrene particles, each with two spheres assembled to form a stable dumbbell (Starodub *et al.*, 2012 ▸). The samples were injected into the X-ray path using an aerosol injector. The ‘intensity profile’ convergence progress is shown in Figs. 4 ▸(*a*) and 4 ▸(*b*), with some representative patterns shown in Fig. 4 ▸(*c*). Note that there are patterns resulting from multiple dumbbell particles. The presented analysis is applicable to this data set, because SAXS analysis does not require that each scattering pattern results from one particle. The monodispersity is the only strict requirement on the samples, meaning that the particles need to be identical and well separated. Fig. 4 ▸(*d*) shows the retrieved models, and Fig. 4 ▸(*e*) shows a comparison of the model profiles with the experimental ‘intensity profile’. Unlike in the nanorice case, *sastbx.shapeup* retrieved three classes of model, as shown in Fig. 4 ▸(*d*). Models 1 and 2 suggest aggregated spheres and dissociated spheres, respectively. The other eight out of ten models are consistent with each other and all are in good agreement with the dumbbell shape. Models 1 and 2 are ranked third and ninth among the ten retrieved models, based on the chi scores (see *Methods* [Sec sec2]). The other eight similar models were averaged to a model represented as model 3 in Fig. 4 ▸(*d*). The low information content of the SAXS profile is the reason for model degeneracy, *i.e.* multiple models may match the same SAXS profile. It is necessary to retrieve multiple models for real-time feedback, because a single model with the lowest chi score could be an incorrect model whose SAXS profile happens to agree with the experimental data. If the model reconstruction is carried out using other approaches, many reconstruction trials should be conducted to obtain multiple models for assessment of consistency.

For the dumbbell data set, the final radius obtained from *sastbx.shapeup* is 89 nm, very close to the expected value of 91 nm (Starodub *et al.*, 2012 ▸). The consistency of the retrieved models with the expected model [reconstructed using the correlation scattering method; see Starodub *et al.* (2012 ▸)] indicates that the proposed analysis approach can be used to guide the model reconstruction. More importantly, the same models can be readily available even during experimental beam time. The real-time feedback will improve the experiment throughput.

### 3.3. Some practical concerns   

As mentioned in the case studies, the convergence of the virtual SAXS profile can be monitored by comparison with a reference SAXS profile, either from *a priori* knowledge or from the merged data of the whole data set. The convergence can also be evaluated by a bootstrapping approach, or by randomly splitting the whole data set into halves and comparing the consistency. It is possible to include an outlier rejection scheme in subsequent analysis by excluding the patterns that have obviously different integrated profiles. Such analysis is not carried out in this report, because the goal of this study is to check whether the merged data can be analysed as SAXS data and whether the retrieval models agree with expectation without invoking a sophisticated screening analysis to avoid model bias. Nevertheless, the outlier rejection should be able to improve the accuracy of the virtual SAXS profile. For example, the fluctuations of the correlation profile shown in Figs. 3 ▸(*b*) and 4 ▸(*b*) are due to the presence of false-positive scattering patterns, which result from unknown particles (either aggregates or water droplets). Such outliers are not major problems for the cases presented in this work, as the correlation coefficients converge to a stable value by including more and more patterns in the virtual SAXS profile. This outlier rejection procedure could take place in the stage of model refinement.

The virtual SAXS profile is valid only if the orientations of the particles are completely random, so the resulting orientation distribution covers the SO3 space uniformly. If the particles have orientation preferences, some weighting schemes have to be implemented, based on the orientation distributions, so that each orientation has a fair representation in the final converged virtual SAXS profile. In some cases, the orientation preference can be utilized to help solve the structures. For example, Elser (2011 ▸) has proposed a method to align molecules on one axis and measure the scattering patterns to extract more information. He suggested to systematically tilt the axis that the molecules are aligned with to obtain scattering information from other orientations; this approach is similar to two-dimensional crystallography.

The model retrieval or reconstruction algorithms do not need particle anisometry information. The SAXS profile is a result of spherical averaging of scattering intensity by sampling intensities from all orientations. In conventional SAXS experiments, this is achieved by collecting intensity from an ensemble of particles. In the virtual ensemble scattering case presented here, the intensities are summed computationally. The *sastbx.shapeup* program finds the model whose SAXS profile has the minimum chi score compared to the experimental data. This is based on the assumption that models are similar if their SAXS profiles are similar (real-space model *versus* reciprocal-space intensity distribution). If more structures are included in the database, the chance of finding a model that is structurally more similar to the sample particle/molecule is higher. Of course, this will be at the cost of a longer search time. Compiling a database with enough model diversity is a trade-off between accuracy and speed.

## 4. Conclusions   

In summary, SAXS profiles can be merged during single-particle scattering experiments at XFEL facilities. The analysis of a SAXS profile, in particular, the fast model retrieval, allows highly plausible models to be examined at near real time. Using this approach, the single-particle scattering data can be quickly evaluated and retrieved models can be used to aid XFEL data analysis. For particles with simple structures, some clues can be obtained by examining individual scattering patterns; as demonstrated, the model retrieval approach can be applied for more complicated models when SAXS data are available. The described procedures and the data sets used in this study are available at http://www.csrc.ac.cn/~HaiguangLiu/.
